# 1304. Antimicrobial Management of *Stenotrophomonas maltophilia* Pneumonia: Does TMP-SMX Dose Affect Treatment Failure?

**DOI:** 10.1093/ofid/ofab466.1496

**Published:** 2021-12-04

**Authors:** J Myles Keck, Nicholson Perkins, Delaney Adams, Mia Warner, Julanne McCommon, Athena L Hobbs

**Affiliations:** 1 Baptist Memorial Hospital-Memphis, Memphis, Tennessee; 2 university of tennessee, Memphis, Tennessee; 3 University of Tennessee College of Pharmacy, Memphis, Tennessee; 4 Methodist University Hospital, Memphis, Tennessee

## Abstract

**Background:**

Limited literature exists to guide the appropriate antimicrobial choice, dose, and duration for *Stenotrophomonas maltophilia* (*S. maltophilia*) pneumonia.

**Methods:**

This multi-center, retrospective cohort study was conducted in adults diagnosed with *S. maltophilia* pneumonia in eight hospitals between March 1, 2014 and August 31, 2020. Patients were enrolled into one of two arms: those treated with TMP-SMX and those treated with alternative antibiotics. The primary outcome was a composite of treatment failure, defined as the need for alternative antibiotics with *in vitro* activity against *S. maltophilia*, isolation of *S. maltophilia* on repeat cultures drawn greater than 72 hours from the index culture, or in-hospital mortality. Other clinical outcomes that were accessed include the TMP-SMX dosing regimen, and 30- and 90-day mortality rates. This study was approved by the local Institutional Review Board (IRB).

**Results:**

Overall, 213 patients were included; 100 (46.9%) received TMP-SMX, and 113 (53.0%) received alternative therapy. Though there was no difference in treatment failure between the two groups, more patients in the “alternative” antibiotic group required mechanical ventilation (57% vs. 31%, respectively; p < 0.001) (Table 1). 74% of patients who received TMP-SMX received a “low-dose” regimen, characterized by < 10 mg/kg/day. When evaluating treatment failure based on “high” or “low-dose” TMP-SMX, there was no difference in treatment failure. However, we noted that physicians were more likely to utilize an alternative agent in addition to TMP-SMX (p=0.001) compared to an alternative agent. When removing this confounder from the composite endpoint, high-dose TMP-SMX was associated with significantly less treatment failure compared to low dose 23% vs. 49%, respectively (p=0.023). The average TMP-SMX dose of patients in the “low-dose” group was 5 + 2.2 mg/kg/day vs. 13.9 + 3.5 mg/kg/day in the “high-dose group. Moreover, when serum creatinine was evaluated at baseline, day 3, day 5, and day 7, no increase was seen in patients who received high dose TMP-SMX (Table 1).

Table 1: Clinical Outcomes

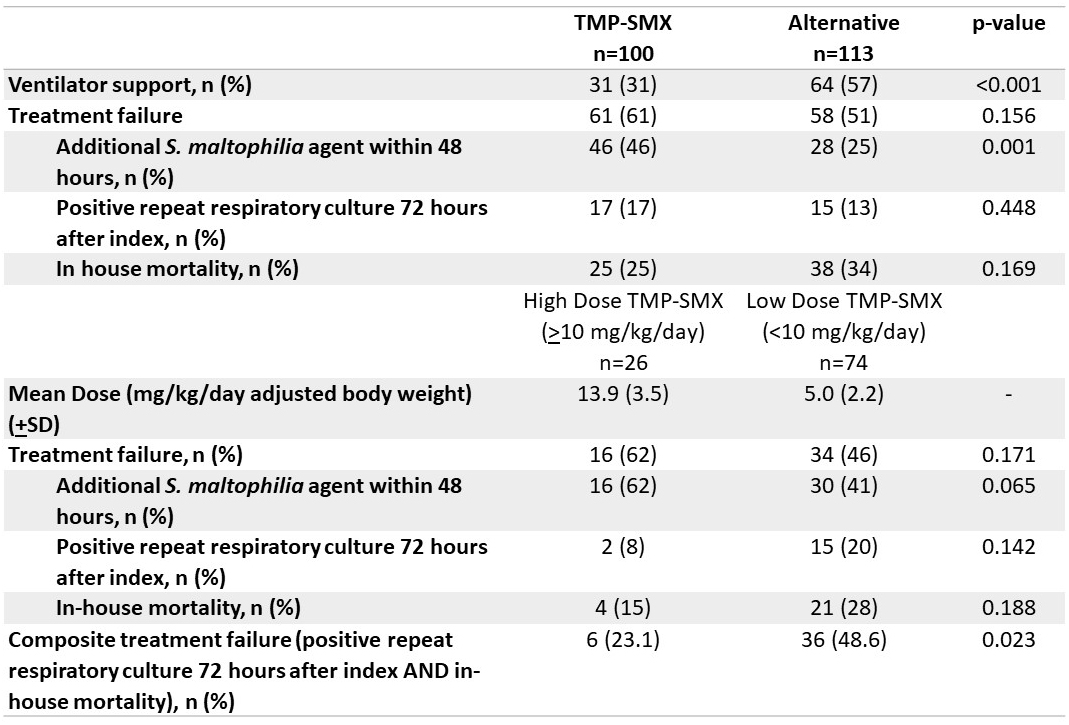

**Conclusion:**

These preliminary results suggest that appropriate dosing of TMP-SMX may be more important for clinical success in patients with *S. maltophilia* pneumonia than the choice of agent.

**Disclosures:**

**All Authors**: No reported disclosures

